# An international comparison of noncommunicable disease reporting: the case of diabetes mellitus

**DOI:** 10.25646/5989

**Published:** 2019-06-27

**Authors:** Lukas Reitzle, Christian Schmidt, Christa Scheidt-Nave, Thomas Ziese

**Affiliations:** Robert Koch Institute, Berlin Department of Epidemiology and Health Monitoring

**Keywords:** HEALTH REPORTING, DISSEMINATION, DIABETES MELLITUS, NCD SURVEILLANCE

## Abstract

Against the background of the growing burden associated with diabetes mellitus, the German Federal Ministry of Health commissioned the Robert Koch Institute to develop a national diabetes surveillance. The periodic publication of up-to-date data needed by diverse target audiences (dissemination) to develop subsequent public health measures is a crucial aspect of disease surveillance. The study produced an overview of diabetes surveillance in various countries with the intention of developing a dissemination strategy. This involved a two-stage process beginning with an online survey of public health experts from 46 countries. Structured Internet research was then carried out for countries that did not provide a response (19 out of 46). The majority of countries (38 out of 46; 83%) include diabetes in their health reporting; three quarters (29 out of 38; 77%) of these countries rely on an indicator-based reporting system. The study found that the most common formats used to publish information about diabetes and other noncommunicable diseases were topic-specific reports (24 out of 36; 67%) and national health reports (23 out of 36; 64%), followed by online formats such as websites or databases (20 out of 36; 57%). Moreover, health reporting primarily targets politicians (19 out of 20; 95%) as well as the media and the press (16 out of 20; 80%). The study found that both printed and online publications form part of a comprehensive dissemination strategy, however address different audiences.

## 1. Introduction

The burden associated with diabetes mellitus and other noncommunicable diseases (NCDs) is steadily increasing in Germany as it is throughout the world [1, 2]. In 2013, the World Health Organization (WHO) adopted the Global Action Plan for the Prevention and Control of NCDs as a means of facing up to the growing challenges posed by noncommunicable diseases [3]. The WHO’s action plan contains six objectives, one of which includes monitoring the trends and determinants of NCDs. This led the German Federal Ministry of Health to commission the Robert Koch Institute (RKI) to set up a diabetes surveillance system that could serve as a pilot project for the surveillance of other noncommunicable diseases in Germany. Together with the project’s scientific advisory board, a framework for the surveillance of diabetes was developed and 40 indicators were defined [4].

Public health surveillance is defined as the continuous, systematic collection, analysis and interpretation of health-related data that is needed for the implementation of public health measures aimed at protecting and promoting the health of the population (‘data for action’) [5]. This definition implies that health monitoring data should be processed in a manner that reflects the needs of its various target audiences. Data from health surveillance can be used to (1) identify high-risk populations, (2) develop prevention strategies, (3) draw up new hypotheses on diseases dynamics, (4) raise awareness about trends and disease-related risk factors, and (5) encourage people to take conscious decisions about their health [6, 7].

In order for health reporting data to be used in this manner, target audiences must be provided with up-to-date information (dissemination). In public health sciences, as in other fields, a gap exists between the production of new knowledge and its translation into practice and policy [8]. This underscores the importance of developing a dissemination strategy as part of the surveillance system that can provide the basis for the development of informed health policy measures [9]. However, it is essential that formats and the communication channels used to disseminate the information reflect the competences and level of expertise that a specific target audience has with regard to a particular aim [10, 11].

The WHO identifies four target audiences as part of its Global Monitoring Framework for the surveillance of non-communicable diseases: (1) healthcare providers, (2) policy makers, (3) service providers, and (4) the general population [12]. In addition, other groups that also need to be addressed include patients, doctors who treat diabetics, and scientists and scientific institutions. The WHO does not state which formats or communication channels should be used to provide NCD-related information, nor does it provide guidance on which information should be provided to the various target audiences. When it comes to formats and communication channels, however, advances in digitisation, in particular, are opening up new ways of visualising and processing data [13]. Moreover, social media and social networks offer further opportunities with which to disseminate health-related information [10, 14].

In addition to building on the experience gained by other public health institutes, best practice examples from other countries are to be used as a model to develop a dissemination strategy for diabetes surveillance at the RKI. An international workshop was held in this context at the RKI in June 2018 that also involved the presentation of innovative formats [15]. Furthermore, a study of health reporting on NCDs was performed using diabetes as an example. The aim of the study was to provide an overview of formats and target audiences of health reporting by the member states of the Organisation for Economic Co-operation and Development (OECD), the European Union (EU), and other selected European countries.

## 2. Methodology

**Figure 1:**
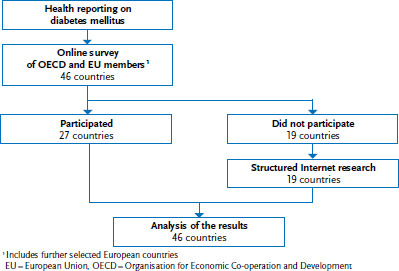
Overview of the process of surveying expert participants and Internet research Own diagram

The study used a two-step approach to collect data about national health reporting of diabetes and noncommunicable diseases (Figure 1). The first step involved surveying national public health experts from the selected countries using an online survey in English. The survey focused on diabetes and NCD-specific health reporting including the formats that were being used and the target audience that was being addressed. The results were supplemented by structured Internet research into countries that did not participate in the expert survey. Finally, examples of best practices from successful health reporting were also selected. The selection was based on a narrative analysis of the results of the online survey and Internet research.

**Infobox 1:**

**An international comparison of noncommunicable disease reporting: the case of diabetes mellitus**

**Data owner:** Robert Koch Institute

**Aim:** To develop an overview of the strategy, content, formats and target audiences of the health reporting of noncommunicable diseases conducted in OECD and EU countries.

**Geographical focus:** 46 OECD or EU member states, as well as other selected European countries

**Study design:** Two-stage process

**►** Online survey of public health experts
**►** Structured Internet research into countries that did not participate in the online survey

**Participants:**

**►** Experts from 27 countries participated in the study
**►** Structured Internet research was carried out for 19 countries

**Study period:** April to September 2018

The survey was limited to OECD and EU member states and other selected European countries; 46 countries were included in the study (Table 1).

### 2.1 Online survey

The online survey of public health experts took place between April and July 2018. The survey used a questionnaire created with the Acuity4 survey software (version 5.5.1.205) from Voxco®. In order to recruit suitable participants, the RKI’s network was used to contact EU and OECD public health institutes as well as those from other European countries. The aim was to ensure that people with expertise in diabetes and health reporting participated in the study. In cases where it was impossible to find someone suitable to answer the questionnaire, a request to participate in the survey was sent to health ministries and national statistical offices. Other institutions were only contacted if they had been named by a member of one of these institutions.

The online survey comprised 39 questions and was divided into two subject areas (Annex Table 1). On the one hand, the questionnaire focused on the framework behind diabetes-specific health reporting, its integration into a diabetes strategy, and the indicators and data sources that were used. On the other hand, it also examined the formats that were being used and the target audiences that health reporting sought to address. Furthermore, respondents were also asked to upload the reports they mentioned during the questionnaire or to provide a link to documents that were available online. After the online survey had been completed, a review was undertaken to ensure that each country had only provided one response. If more than one survey existed for a country, the data provided on these questionnaires were merged.

### 2.2 Structured Internet research

Between August and September 2018, structured Internet research was conducted into countries that had not supplied any data by the end of the online survey period (July 2018). The analysis focused on the country’s framework, indicators, and the formats used for diabetes and NCD-specific health reporting. The Internet research was carried out in the following manner: first, a search was conducted of the websites of the respective national public health institutes, health ministries and statistical offices for keywords linked to diabetes and noncommunicable diseases. The Google search engine was then used to search for a combination of terms. In each case, the search term consisted of either diabetes, noncommunicable disease or NCD, alongside surveillance, monitoring, strategy, report, health reporting or indicators. Lastly, the respective country name (in English) was added to the search term. The study then examined the first 30 search results. Public health institutes, ministries of health or statistical institutes do not always provide relevant information in English, German or French. In these cases, their websites were translated into English using Google Translate and the resulting translations were searched for the keywords mentioned above.

The research was limited to the framework (strategy, indicators, data sources) and formats (reports, websites, databases) used by the country for health reporting. Furthermore, only reports and formats published in or after 2000 were included in the study. Unfortunately, it was impossible to identify the reports’ target audiences as the websites that published them provided no relevant information about this issue.

## 3. Results

Of the 46 countries included in the overall study, 27 (59%) participated in the online survey (Figure 1). The majority of participants were from public health institutes (20 out of 27; 74%) and, albeit less frequently, from health ministries (5 out of 27; 19%). Structured Internet research was carried out for the remaining 19 countries so that diabetes-specific health reporting could be evaluated for all 46 countries included in the study.

**Figure 2:**
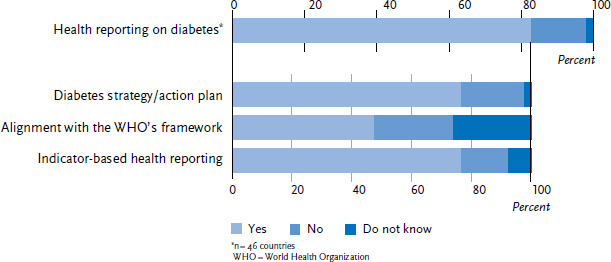
Concept of diabetes-specific health reporting (n=38 countries) Source: An international comparison of noncommunicable disease reporting: the case of diabetes mellitus

The first part of the study focused on the framework employed for diabetes health reporting and the indicators used to depict developments in the course of the disease. In total, four out of five countries include diabetes mellitus in their national health reporting (Figure 2). Of these, over three quarters have defined a national diabetes strategy or action plan. In addition, half of the countries that conduct diabetes health reporting state that they follow the WHO’s NCD Global Monitoring Framework [16]. However, no conclusions could be made about ten of the 38 (26%) countries. The majority of countries (29 out of 38; 77%) use an indicator-based system for reporting diabetes, with eight countries using a system exclusively for diabetes mellitus and 21 countries including diabetes in their surveillance of non-communicable diseases.

**Figure 3:**
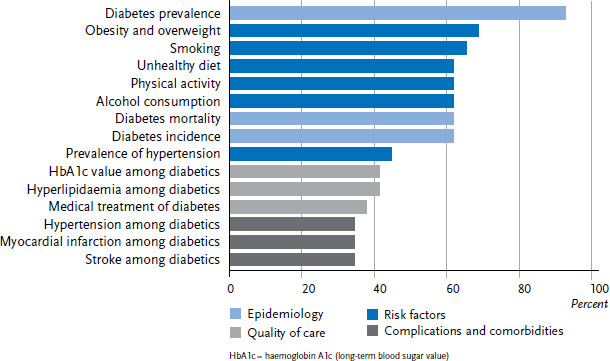
The 15 most commonly used indicators in diabetes mellitus surveillance (n=29 countries) Source: An international comparison of noncommunicable disease reporting: the case of diabetes mellitus

The evaluation of the indicators determined by the study led to the identification of 142 different indicators or indicator clusters that are used in diabetes surveillance. These were divided into the following six areas: epidemiology, disease burden, complications and comorbidities, risk factors, quality of care, and public health measures. The 15 most common indicators are shown in Figure 3. The ranking demonstrates that most countries use epidemiological indicators such as incidence, prevalence and mortality of diabetes as well as behavioural risk factors. Indicators covering quality of care, complications and comorbidities were reported less frequently. In 23 out of 28 countries (82%), the indicators relied on data sources that included regularly conducted national health surveys. Routine data, such as claims data from hospitals and medical practices, insurance data and data from other institutions, are included in diabetes health reporting in 19 out of 28 (68%) countries.

**Figure 4:**
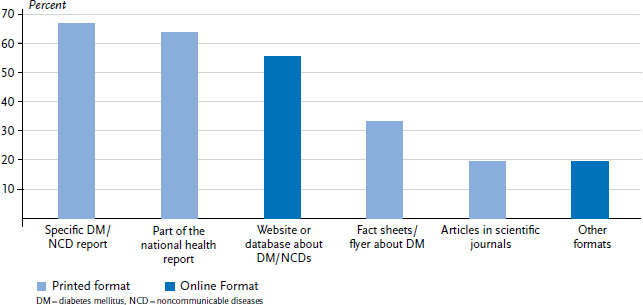
Formats used in diabetes health reporting (n=36 countries) Source: An international comparison of noncommunicable disease reporting: the case of diabetes mellitus

In addition to questions about the framework governing diabetes surveillance, the study focused on the formats used and the intended audience of diabetes-specific health reporting. The study found that different formats were being used to deliver the results (Figure 4). These can be divided into printed formats (including digital formats in print layouts such as Word and PDF) and formats that were only available online (web pages and databases). The study evaluated a total of 67 reports, 25 online formats and nine other formats from 36 countries. These were either provided as links, uploaded by the respondents, or were found during the Internet research. Due to the common ground covered by the reports and for reasons of clarity, health reports on diabetes mellitus (DM) and on noncommunicable diseases (NCD) were summarised as DM/NCD reports. Two thirds of countries (24 out of 36; 67%) publish their results in a specific report on diabetes or together with other noncommunicable diseases. Furthermore, results are often included in countries’ interdisciplinary national health reports. Flyers and fact sheets (12 out of 36; 33%) as well as scientific publications (7 out of 36; 19%) are used to a much lesser extent. In addition to traditional printed formats, results in 20 out of 36 (56%) countries are provided on a website or online database, with 9 out of 36 (25%) countries providing a database that enables the results to be queried directly. More than half of the websites and online databases included tools for interactive data visualisation. Furthermore, 7 out of 36 (19%) countries use other formats to publish results. These are mainly newer formats related to social media such as Twitter, Facebook or YouTube, but also include press releases. According to the respondents, these formats are primarily used to draw attention to the issue of diabetes and to raise awareness in society about this health problem.

Reports, flyers, websites and online databases are generally made available in a country’s own language. In about half of the cases (48 out of 92; 52%), they are also available in English. However, English is an official language in seven of these countries. Most formats (67 out of 92; 73%) are published or updated regularly. The majority of reports, websites and databases are updated once a year (35 out of 67; 52%) or within two to five years (28 out of 67; 42%).

**Figure 5:**
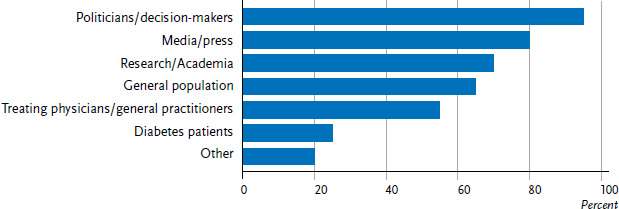
Target audiences of diabetes-specific health reporting (n=20 countries) Source: An international comparison of noncommunicable disease reporting: the case of diabetes mellitus

The public health experts were also asked about the target audiences that diabetes-specific health reporting was seeking to address (Figure 5). Almost all of the countries surveyed primarily target policy and decision-makers in the health sector, followed by the media and the press, and scientists and the general population. Slightly more than half of the surveyed countries list doctors who treat diabetics as their target audience. Diabetes patients are specifically addressed by just one in four countries. These results are based entirely on the data gathered from the countries that participated in the online survey; no information was available about the audience targeted by the documents identified through Internet research. In total, 20 countries provided information about the audiences that their formats were seeking to address (seven countries provided no information about this at all).

**Figure 6:**
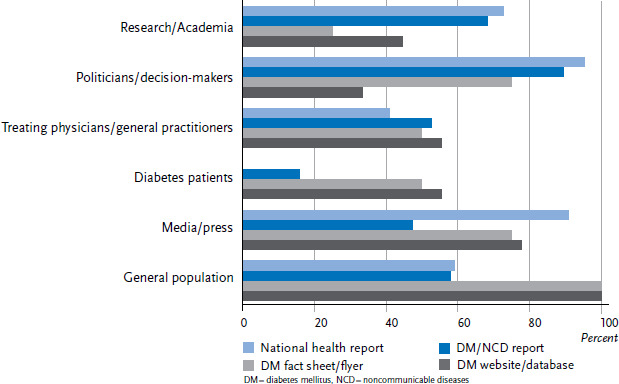
Presentation addressees depending on the format used for diabetes-specific health reporting (n=54 formats) Source: An international comparison of noncommunicable disease reporting: the case of diabetes mellitus

Different formats are used to address different audiences and, as such, they were evaluated with respect to their intended audience (Figure 6). However, since articles in scientific journals are primarily aimed at scientists, and the category ‘other formats’ contained a diverse range of formats, neither was included in the overall evaluation. Health sector professionals had provided information about the target audiences of 54 reports, all of which were then used in the study. The analysis demonstrated that interdisciplinary national health reports primarily target healthcare policy and decision-makers (21 out of 22; 96%) and the media and the press (20 out of 22; 91%). However, researchers (16 out of 22; 73%) and the general population (13 out of 22; 59%) were addressed in more than half of these cases. A similar picture emerges for topic-specific health reports on diabetes and noncommunicable diseases. Just under half of these reports target the media and the press. In contrast, flyers and fact sheets on diabetes were primarily aimed at the general population (4 out of 4) and, to a lesser extent, at political decision-makers and the media (3 out of 4). In addition, treating physicians as well as diabetes patients themselves are also named as target audiences in these cases (2 out of 4). Online formats (mainly websites) were aimed at the general population (9 out of 9) as well as the media and the press (7 out of 9). However, some online formats were also directed at diabetes patients (5 out of 9; 57%) and their physicians (5 out of 9).

This results in a clear picture: whereas political decision-makers are particularly targeted by interdisciplinary and topic-specific health reports, online formats, flyers and fact sheets are mainly used to address the general population. The media and the press, as the second most commonly mentioned target audiences, are addressed via both more traditional printed publications and more modern online formats.

The study also sought to select examples of best practices from the formats identified by the online survey and the Internet research (Table 2). Ultimately, the study identified print and online publications from four countries that the authors view as having successfully implemented various aspects of health reporting.

## 4. Discussion

The online survey of public health experts and the Internet research enabled a structured overview of diabetes-specific health reporting in the EU and OECD countries to be developed. The majority of countries under study include diabetes mellitus in their national health reporting and have established indicators for disease surveillance. Health reports that were either printed or typeset and published online were the most commonly used formats. Online formats such as websites and databases are used in more than half of the countries under study, some of which also provide innovative visualisation tools. All of the countries principally targeted policy makers, followed by the media and press, individuals and institutions involved in public health research, and the general population.

The literature provides very little information that could offer an overview of the surveillance systems used to collect data on noncommunicable diseases in various countries. However, as part of its action plan for the prevention and control of NCDs, the WHO regularly reports on the progress that each member state is making towards reaching the action plan’s objectives, and also publishes relevant documents [17]. The proportion of countries with an identified diabetes strategy is comparable to the results of the online survey. The few exceptions that did occur were due to the fact that five cases from the online survey involved general NCD strategies that only implicitly included diabetes.

Although the WHO Framework [16] recommends that countries monitor behavioural risk factors (alcohol consumption, tobacco consumption, physical inactivity, obesity and overweight, unhealthy dietary habits) as part of their disease surveillance, the study found that only half of countries do so. However, closer analysis of the indicators demonstrates that two thirds of indicator systems include behavioural risk factors as indicators, and, thus, the majority of indicator systems do indeed reflect the WHO’s approach. Moreover, the majority of countries also use data sources that contain both primary and secondary data. Diabetes surveillance at the RKI also includes behavioural risk factors among its indicators [4] as well as data from health surveys and routine data to map the dynamics of the disease. Information about the strengths and weaknesses as well as the opportunities offered by these data sources can be found in this issue of the Journal of Health Monitoring in the contributions entitled Social inequality and diabetes mellitus and secondary data in diabetes surveillance.

At the same time, the literature also provides very few recommendations or reviews of the formats and communication channels that are used for health reporting in the context of diabetes and other NCDs. As part of its framework, the WHO recommends the publication of fact sheets and data books (comprehensive volumes containing tables) that are entirely focused on the data [12] and that only take the requirements of target audiences with regard to format and data processing into account to a limited extent.

The study found that traditional printed reports are the most common publications used to provide information from disease surveillance. However, websites and online databases also offer a variety of options for data visualisation and are already being used by some countries to present the results from the surveillance of noncommunicable diseases. These tools are already being used to a greater extent in the surveillance of infectious diseases [18]. For example, the AIDSVu project uses regional analyses to identify vulnerable groups and subsequently implement targeted public health interventions in the most affected communities [19]. This approach could also open up diverse opportunities for NCD surveillance. For example, the Fingertips platform operated by Public Health England (Table 2) provides detailed analyses of various diabetes indicators that are used at the regional level to plan public health measures [15]. In addition, social media offer new opportunities to provide information to the public [10] and are already being used in some countries. In these cases, the focus is on raising awareness about diseases such as diabetes and their related risk factors. Similarly, the CDC (Centers for Disease Control and Prevention) in the United States of America have developed infographics that provide information about diabetes to the general public (Table 2). However, more research is needed into the effectiveness of public health interventions via social media [14].

The online survey found that diabetes reporting primarily seeks to address policymakers and health-care decision-makers, followed by the media and press. The WHO also identifies policymakers and health-care decision-makers as two of its four target audiences for information from NCD surveillance in addition to service providers and the general population [12]. The literature also emphasises the importance of addressing service providers and the general public during the establishment of public health surveillance systems [6, 7, 10]. Whereas diabetes surveillance tends to target politicians with the aim of encouraging the development of interventions and other public health measures (data-based decision-making) [20], the primary purpose of providing information to the general public is to raise awareness about diseases and risk factors as well as to provide other information that is relevant to public health [7]. The media and the press can serve as important disseminators of information aimed at the general public and, therefore, indirectly raise awareness among politicians of the importance of specific topics [9]. However, a discussion is currently taking place in the literature about the effectiveness of the media in terms of its impact on politics; as of this time, the impact of the media is yet to have been unequivocally proven [21]. Moreover, personal contact, which can take place during individual meetings and symposia, is particularly important when it comes to addressing politicians [10]. In summary, it is crucial that the needs of different target audiences are considered [22, 23] and that indicators are used to review the use of the formats provided [10, 24, 25].

### 4.1 Limitations

The two-step approach and the use of two different methods to collect data on diabetes-specific health reporting means the study faces a number of limitations. As some countries did not participate in the online survey, the only information and documents available in these cases were those that were found on the Internet. These were available in English, German or French or via Google Translate. Moreover, as data about target audiences are not freely available and can only be supplied by public health experts, the Internet research was unable to provide information about the intended target audiences in these cases. Similarly, as no information was available about the people who were actually using the respective formats, it was impossible to judge whether the intended audiences were actually being reached. Furthermore, the study was limited to publications by state institutions and ministries; publications by non-governmental organisations, such as patient associations or professional associations, were not included in the analyses. Equally, the study’s focus on national health reporting meant that regional reports or other regional formats were not considered. Finally, it is unclear whether the results of the study can be applied to all noncommunicable diseases, as diabetes receives greater attention than other diseases.

### 4.2 Conclusion and outlook

Public health surveillance systems provide the data required to make health policy decisions and to establish public health policies. The growing complexity caused by the increasing number of data sources and findings from scientific research means that information needs to be provided transparently and understandably. In addition to more traditional formats, such as reports, new tools for visualisation and interactive databases can enable data to be depicted in a manner that is understandable and which facilitates access to different target audiences.

Health reports about NCDs primarily address politicians and health policy decision-makers. However, these individuals can be reached in different ways. In addition to reports, established communication tools and social media also provide appropriate communication channels that can raise the priority and awareness of specific public health challenges. Discussions and symposia also constitute an important aspect of dissemination strategies.

The study’s findings are to be used to develop a dissemination strategy for diabetes surveillance at the RKI. Diabetes surveillance particularly targets politicians and decision-makers in the healthcare sector. In line with the results of this study, a diabetes report is to be drawn up for this target audience. In addition, information is also to be provided to the general public and the media with the aim of raising awareness about the growing challenges posed by noncommunicable diseases. In order to address these audiences, a website is to be developed to provide visual representation of the results of diabetes surveillance; social media, such as Twitter and YouTube, are also to be used more regularly. The examples of best practices, which were collected during the survey and Internet research, will provide an important basis for the further development of these formats.

## Key statements

- 83% of countries include diabetes in their national health reporting.
- 77% of countries use an indicator-based surveillance system for health reporting on diabetes.
- Topic-specific and general health reports (67%) are the most commonly used formats to publish information about diabetes.
- 56% of countries use websites or online databases to provide information about diabetes.
- The study’s participants maintained that policy and decision-makers in the healthcare sector were the most important target audiences of diabetes health reporting.

## Figures and Tables

**Table 1:** Countries included in the survey and Internet research Own table

OECD	OECD and EU		EU	Other countries
Australia	Belgium	Austria	Bulgaria	Albania
Canada	Denmark	Czech Republic	Croatia	Liechtenstein
Chile	Estonia	Hungary	Cyprus	Montenegro
Iceland	Finland	Poland	Malta	North Macedonia
Israel	France	Portugal	Romania	Serbia
Japan	Germany	Slovakia		
Mexico	Greece	Slovenia		
New Zealand	Ireland	Spain		
Norway	Italy	Sweden		
South Korea	Latvia	The Netherlands		
Switzerland	Lithuania	United Kingdom		
Turkey	Luxemburg			
United States of America				

EU = European Union, OECD = Organisation for Economic Co-operation and Development

**Table 2:** Best practice examples from diabetes health reporting Own table

	Interactive graphics	Flexible data analysis	An informative, illustrated health report	An informative flyer for social media use
**Institute**	Federal Office of Public Health	Public Health England	National Institute for Public Health and the Environment	Centers for Disease Control and Prevention
**Country**	Switzerland	United Kingdom	The Netherlands	United States of America
**Special feature**	User-friendly and nuanced graphical depiction of various indicators	Platform enabling a flexible visualisation of data on diabetes and a variety of options for evaluation	A well-structured report on the development of health in the Netherlands. Implements a balanced design using text and illustrations	Informative infographics and innovative formats involving social media aimed at the general population
**Format**	Website	Database/website	Report	Flyer/social media
**Link**	https://www.obsan.admin.ch/de/MonAM	https://fingertips.phe.org.uk/	https://www.rivm.nl/publicaties/volksgezondheid-toekomst-verkenning-2018-gezond-vooruitzichtsynthese	https://www.cdc.gov/diabetes/library/socialMedia/index.html

## Annex Table 1 Online survey 'Health reporting on diabetes mellitus' Own table

### 1. Introduction

**1.1 Would you like to participate in this survey?**
  **1:** Yes
  **2:** No
**1.2 For which country do you answer this questionnaire?**
  *Please enter the country here*
**1.3 What kind of institution are you working for?**
  **1:** Ministry
  **2:** National public health institute
  **3:** Regional or local public health institute
  **4:** Other governmental institution
  **5:** University
  **6:** Research institute
  **7:** Health insurance body
  **8:** Think tank, private consultancy
  **9:** NGO, association or interest organization
  **10:** Medical care facility
  **11:** Social care facility
  **12:** Others, namely:
  **13:** No reply
**1.4 What is the name of the institution you are working for?**
  *Text field*
  No reply

### 2. Diabetes mellitus surveillance

**2.1 Do you know whether there is a national health reporting on diabetes mellitus in place in your country?**
  **1:** Yes
  **2:** No
  **3:** Do not know
**2.2 Does the national diabetes reporting follow the WHO framework for surveillance of noncommunicable diseases?**
  **Table 1:** Framework for national NCD surveillance
  **Exposures** Behavioural risk factors: *tobacco use, physical inactivity, the harmful use of alcohol and unhealthy diet.* Physiological and metabolic risk factors: *raised blood pressure, overweight/obesity, raised blood glucose, and raised cholesterol.* Social determinants: *educational level, household income, and access to health care.* **Outcomes** Mortality: *NCD-specific mortality.* Morbidity: *Cancer incidence and type (as core).* **Health system capacity and response** Interventions and health system capacity: *infrastructure, policies and plans, access to key health-care interventions and treatments, and partnerships.*
  Surveillance of Noncommunicable Diseases. Report of a WHO Meeting. *Geneva, World Health Organization, 2010.*
  **1:** Yes
  **2:** No
  **3:** Do not know
**2.3 Has there been a national diabetes strategy (action plans or health targets) developed in your country?**
  **1:** Yes
  **2:** No
  **3:** Do not know
**2.3.1 Is the national diabetes strategy (action plans or health targets) available in English?**
  **1:** Yes
  **2:** No
  **3:** Do not know
**2.3.2 Please enter the corresponding internet link/ URL address or upload the national diabetes strategy here.**
  *Text field*
**2.4 Is there an established set of health-related indicators (social and environmental determinants, risk factors, health-related outcomes) in your country, which is used for health reporting of noncommunicable disease and/or diabetes mellitus?**
  **1:** Yes, for noncommunicable disease including diabetes mellitus
  **2:** Yes, specifically for diabetes mellitus only
  **3:** No
  **4:** Do not know
**2.4.1 Please enter the corresponding internet link/URL or upload the document of the indicator system for health reporting on diabetes mellitus or NCDs in your country here.**
  *Text field*
**2.4.2 Do you distinguish within your set of health-related indicators between core indicators and additional indicators?**
  **1:** Yes, we have defined a subset core indicators
  **2:** No, we do not distinguish within the set of indicators
  **3:** Do not know
**2.4.3 How many core indicators for diabetes mellitus have you defined?**
  *Text field*
  Do not know

### 3. Formats

**3.1 What formats are used for publication of health reports covering diabetes mellitus in your country at national level?**
*Multiple answers allowed*
**Information on diabetes mellitus is published…**
  **1:** … as specific chapter in a comprehensive national health report.
  **2:** … as part of a comprehensive report on noncommunicable diseases.
  **3:** … as comprehensive report solely on diabetes mellitus.
  **4:** … as short report on diabetes mellitus.
  **5:** … as fact sheet/flyer.
  **6:** … as publication in a peer-reviewed journal.
  **7:** … as statistical online-database.
  **8:** … as main topic on the website of a national public health institute or another institution.
  **9:** … as another report format.
  **10:** Do not know
**3.2 For the health reports including diabetes mellitus you know, please name the …**
  **- Title**
  **- Type of report**
  **- Publishing institution**
  **- Year of publication/Regular publication**
  *In case of regularly published national health reports please list only the latest issue.*
  *If the reports are available in English we invite you to upload the document in a later step.*
  **Table:**
  	Title of the report	Type of report	Publishing institution	Report is regularly published		Year of publication (latest issue)	Report available in English	
  				Yes	No		Yes	No
  1								
  2								
  …								
**3.2.1 For the reports, which are published regularly, could you please indicate the publication frequency?**
**Publication frequency** (<1 year, annually, biannually, every 3-5 year, every 5-10 year, >10 year)
  Report 1
  …
**3.2.2 For the reports you have mentioned, could you please indicate the target audience?**
  *Multiple answers allowed*
  **Table:**
  Report title	Research/academia	Politicians/decision makers	Treating physicians/GPs	Diabetes patients	Media/press	General population	Other	Do not know
  Report 1								
  Report 2								
  …								

### 4. Database (only if applicable)

**4.1 You have indicated that surveillance data on diabetes mellitus is part of an online database. What is the name of the online database?**
  *Text field*
**4.2 Which institution hosts the database?**
  *Text field*
**4.3 Is the database available in English?**
  **1:** Yes
  **2:** No
  **3:** Do not know
**4.4 Does the database include a tool for regional visualization, e.g. an interactive map showing different indicators like prevalence by region within the country?**
  **1:** Yes
  **2:** No
  **3:** Do not know
**4.5 How frequently is the information of the database updated?**
  **1:** Regularly - Please indicate timeframe in years___
  **2:** Only irregular updates
  **3:** Do not know
**4.6 Is the database publically available?**
  **1:** Yes - Please indicate the link/URL__________
  **2:** No
  **3:** Do not know

### 5. Website (only if applicable)

**5.1 You mentioned that diabetes mellitus is a main topic of the website of a national public health institute or another institution in your country. What is the name and link/URL of the website?**
  *Text field*
**5.2 Which institution hosts the website?**
  *Text field*
**5.3 Is the website available in English?**
  **1:** Yes
  **2:** No
  **3:** Do not know
**5.4 Who is the target audience for the website publishing information on diabetes surveillance?**
  *Multiple answers allowed*
  **Table:**
  	1 Research/academia	2 Politicians	3 Treating physicians/GPs	4 Diabetes patients	5 Media/press	6 General population	7 Other	8 Do not know
  Website								
**5.5 Does the website include a tool for regional visualization of data on diabetes mellitus, e.g. an interactive map showing different indicators like prevalence by region within the country?**
  **1:** Yes
  **2:** No
  **3:** Do not know
**5.6 How frequently is the information of the website updated?**
  **1:** Regularly - Please indicate timeframe in months____________
  **2:** Only irregular updates
  **3:** Do not know

### 6. Other format (only if applicable)

**6.1 You have indicated that health information on diabetes mellitus is published in a format other than those listed. Could you please describe the format in more detail (print vs. online, content of the format, etc.)?**
  *Text field*
**6.2 Which institution publishes this format?**
  *Text field*
**6.3 Who is the target audience for this format?**
  *Multiple answers allowed*
  **Table:**
  	1 Research/academia	2 Politicians	3 Treating physicians/GPs	4 Diabetes patients	5 Media/press	6 General population	7 Other	8 Do not know
**6.4 Is this format published regularly?**
  **1:** Yes
  **2:** No
  **3:** Do not know
**6.5 Is this format on diabetes mellitus available in English?**
  **1:** Yes
  **2:** No
  **3:** Do not know
**6.6 Please enter the corresponding internet link/URL address or upload the diabetes mellitus report format here.**
  *Text field*

### 7. Data Sources

**7.1 You have indicated that in your country there is an indicator system for the monitoring of diabetes mellitus in place. Which data sources are you using to collect this data?**
*Multiple answers allowed*
  **Primary data using…**
    1: … a national health survey **specifically on diabetes mellitus**.
    2: … a national health survey on **noncommunicable diseases** including diabetes mellitus.
    3: … a general national health survey covering several topics including diabetes mellitus.
  **Secondary data using**
    4: … other institutions/ministries, namely_______
    5: … data from insurance companies
    6: … data from hospitals/doctors
  **Other**
    7: … Other sources, namely________
    8: Do not know
**7.2 What is the name of the health survey covering diabetes mellitus?**
  *Text field*

### 8. Closing

**8.1 If you know of a relevant health report on the national, sub-national or international level which you consider a good-practice-model for reporting on noncommunicable diseases or diabetes mellitus, please indicate the internet link/URL address and/or upload the report here.**
  *Text field*
**8.2 Do you have any further comments about health reporting on diabetes mellitus or this survey?**
  *Text field*

**Thank you for your participation!**
